# Beneficial effects of walnut (*Juglans regia* L.) oil-derived polyunsaturated fatty acid prevents a prooxidant status and hyperlipidemia in pregnant rats with diabetes

**DOI:** 10.1186/s12986-020-00514-3

**Published:** 2020-10-23

**Authors:** Bingmei Sun, Hua Yan, Chao Li, Linlin Yin, Fei Li, Lianxiang Zhou, Xiuqing Han

**Affiliations:** Department of Gynaecology and Obstetrics, Central Hospital of Linyi, No. 17 Health Road of Yishui County, Linyi City, 276400 China

**Keywords:** Walnut oil-derived PUFA, Dyslipidemia, Oxidative stress, Gestational diabetes

## Abstract

**Background:**

Gestational diabetes mellitus has a long-term effect on pregnant women. Walnut (*Juglans regia* L.) oil-derived polyunsaturated fatty acid (PUFA) possesses multifarious pharmacological activities. This study investigated the beneficial effects of walnut oil-derived PUFA on glucose metabolism, pregnancy outcomes, oxidative stress, and lipid metabolism in gestational diabetes mellitus.

**Methods:**

The GDM rat model was generated by intraperitoneal injection of streptozotocin (40 mg/kg) on gestational day (GD) 6, GD7 and GD8. The differences between groups were estimated using one-way ANOVA followed by the Tukey’s multiple comparison test for post-hoc analysis.

**Results:**

The results indicated that PUFA could mitigate GDM in pregnant diabetic rats, as embodied by the decrease of fasting blood glucose and the increase of plasma insulin and hepatic glycogen levels. Also, PUFA could suppress oxidative stress in pregnant diabetic rats, as reflected by the decrease of malondialdehyde content, an increase of superoxide dismutase, catalase and gutathione peroxidase activities. PUFA could also mitigate the abnormal changes of lipid profiles in plasma and hepatic tissue. Moreover, the relative mRNA expression of sterol regulatory element-binding transcription factor-1, stearoyl-CoA desaturase-1, fatty acid synthase, and acetyl-coenzyme A carboxylase, was suppressed by PUFA in pregnant diabetic rats.

**Conclusions:**

These results suggested that PUFA supplementation during pregnancy is beneficial in preventing diabetic complications in pregnant rats.

## Introduction

Gestational diabetes mellitus, a frequent metabolic disorder in pregnancy, being present in 1–18% of all pregnancies [1]. It has been defined as gestational diabetes mellitus is characterized by hyperglycemia or glucose intolerance with onset during pregnancy resulting from defects in insulin secretion or insulin action [2]. The chronic hyperglycemia of pregnancies is associated with long-term dysfunction and damage of different organs for the mother and offspring as well as a possibility of increased fetal-maternal morbidity [3]. Also, the offspring of women with gestational diabetes mellitus are more likely to develop obesity, impaired glucose tolerance and metabolic disorders in later life [4]. Previous study has reported that the intrauterine oxidative stress environment contributed to adverse outcomes and metabolic diseases influenced fetal programming in pregnancies [5]. Superfluous oxidative stress has been implicated in the pathogenesis and development of gestational diabetes mellitus [6]. Although the underlying molecular mechanisms of diabetes complications are complicated and remain unclear, however preclinical and clinical researches indicated that gestational diabetes is related to oxidative stress, leading to a decline in the antioxidant defense system and an increased production of reactive oxygen species [7, 8]. Thus, it has been speculated that the usage of the antioxidant agent may exert a protective effect against diabetes complications via suppression of reactive oxygen species. Therefore, it is urgent to develop a novel treatment for gestational diabetes.

The usage of traditional medicine and food derived from natural antioxidants is regarded as an alternative therapy for improving oxidative stress in diabetes [9–11]. The green husks, leaves, and seeds of the walnut (*Juglans regia* L.) are the main source of these functional ingredients which have been used as folk medicine for the prevention and treatment of some diseases including anorexia, diabetes mellitus, cancer, thyroid dysfunction and infectious diseases [12]. Polyunsaturated fatty acids (PUFA), phenolic acids, and flavonoids are considered as major active compounds in *Juglans regia* L. seeds [12]. Recently, clinical researches have indicated that the addition of walnut oil in the daily diet may serve as a helpful remedy for patients with diabetes mellitus type 2 [13, 14]. However, whether walnut oil-derived PUFA exhibited beneficial effects against gestational diabetes mellitus is not yet clear.

In the present study, we aim to investigate the beneficial effects of walnut oil-derived PUFA on glucose metabolism, pregnancy outcomes, oxidative stress, and lipid metabolism in gestational diabetes mellitus.

## Materials and methods

### Plant material collection, samples preparation and analysis of PUFA

Walnut (*Juglans regia* L.) nuts were purchased from the local market in Shandong Province and harvested in October 2019 from Nanshan District, Jinan City located in Shandong Province. Walnut oil was extracted from Shandong walnuts according to the cold-press method with minor modification [15]. The preparation process of PUFA is based on previous literature [16] and prepared by a professional following a standard protocol. PUFA is stored at − 20 °C to prevent oxidation and contamination. And free fatty acids were separated and determined by HPLC–UV equipped with Lichrosorb RP-18 column (particle size 5 µm; 150 × 4.6 mm, Merck). The HPLC conditions were as follows: The column temperature was maintained at 30 °C with a flow rate of 0.9 mL/min, the UV absorption at a wavelength of 192 nm and the mobile phase composition was water/acetonitrile (1:9) isocratic for 15 min. The free fatty acid peaks were identified by matching them with fatty acid standards (Sigma-Aldrich, USA). All other chemical reagents were purchased from Aladdin Reagent Co. (Shanghai, China).

### Animals

Animal procedures in this experiment were approved by the Animal Care and Use Committee of the Central hospital of Linyi, by the guiding principles for the care and use of animals published by the National Institute of Health. Wistar rats (10 weeks old female rats: 180–240 g; adult male rats: 300–340 g) were purchased from the Experimental Animal Center of Shandong Province. All rats were housed in a controlled room with a temperature of (22–24 ˚C), a relative humidity of (40–60%) with a light cycle (12/12 h light/dark) and fed with a basic diet and water. After 7 days of adaptation, female rats and male rats were permitted to mate. Pregnancy was confirmed by the presence of a copulatory plug in the next morning, and the day was defined as gestational day (GD) 0.

### Experimental protocol

The GDM rat model was induced by intraperitoneal injection of 40 mg/kg streptozotocin (dissolved in 0.1 mol/L citrate buffer, pH 4.5) on GD6, GD7, and GD8, respectively [17]. Non-diabetic control rats received an equal volume of citrate buffer by intraperitoneal injection. The rats with a fasting blood glucose value of more than 16.7 mmol/L were considered diabetic and used for further researches. The present study was performed in five groups of eight pregnant rats each: Pregnant control group (PC), rats which received the 1% carboxymethylcellulose sodium (CMC) solution by oral gavage. Gestational diabetes mellitus group (GDM), GDM rats which received the 1% CMC solution by oral gavage. LPUFA group, GDM rats which received a low dose of polyunsaturated fatty acids (225 mg/kg body weight, LPUFA). MPUFA group, GDM rats which received a middle dose of polyunsaturated fatty acids (450 mg/kg body weight, MPUFA). HPUFA group, GDM rats which received a high dose of polyunsaturated fatty acids (900 mg/kg body weight, HPUFA). PUFA was dissolved in 1% CMC solution and administered to rats by oral gavage per day from GD 0 to GD 17. The doses of PUFA were selected according to previous study [18] and our pilot study. The pilot study was performed and results indicated that 900 mg/kg could be the appropriate dose to exert hypoglycemic effect in the present study.

### Blood and tissue samples collection

On GD 18, rats were euthanized by light ether anesthesia after overnight fasting, the fetuses, placentas, and liver tissues were immediately weighed and stored at -80 °C until further assay. Blood samples were collected from the orbital venous plexus. The hemoglobin (Hb) and glycated hemoglobin (HbA1c) levels were measured in whole blood sample. Plasma was immediately obtained from blood after centrifugation at 10,000 rpm for 15 min at 4 °C and used for mesurment of plasma glucose, insulin, triglycerides (TG), total cholesterol (TC), low-density lipoprotein cholesterol (LDL-C), and high-density lipoprotein cholesterol (HDL-C). The hepatic tissues were minced and homogenized in an ice-cold saline solution. After that, the hepatic homogenates were centrifuged at 10,000 rpm for 20 min at 4 °C. The supernatant was collected for further assays.

### Determination of fasting insulin concentration, fasting blood glucose levels, Hb, and HbA1c

Body weight, serum glucose, and insulin levels were assayed on GD 0, GD 9, and GD 18. The blood samples were obtained from the orbital venous plexus after overnight fasting. Plasma samples were obtained from blood after centrifugation at 10,000 rpm for 15 min at 4 °C. Insulin concentration was measured by a rat insulin enzyme-linked immunosorbent assay (ELISA) kit (Thermo Scientific). Blood glucose, Hb, and HbA1c levels were measured using commercial kits obtained from Nanjing Jiancheng Bioengineering Institute (Jiangsu, China) according to the supplier’s instructions. Measurement of Homeostasis Model of Insulin Resistance (HOMA-IR) was assayed by the following formula: HOMA-IR = (Fasting blood glucose × fasting insulin)/22.5

### Oral glucose tolerance test (OGTT) and intraperitoneal insulin tolerance test (IPTT)

OGTT and IPTT were measured on GD 17. Before the measurement, all rats were fasted overnight. For OGTT, rats were orally administered with glucose at 2 g/kg body weight. For IPTT, rats were intraperitoneally injected with insulin at 2 units/kg body weight. Blood glucose concentrations were assayed from the orbital venous plexus at baseline and after the glucose and insulin loading (30, 60, 90, and 120 min).

### Estimation of hepatic glycogen levels and oxidative stress

Hepatic tissues were homogenized in an ice-cold saline solution. After that, the hepatic homogenates were centrifuged at 10,000 rpm for 20 min at 4 °C. The supernatant was collected for further assays. Hepatic glycogen levels were assayed with commercial kits obtained from Nanjing Jiancheng Bioengineering Institute (Jiangsu, China) according to the supplier’s protocols. The oxidative stress was also examined by the measurement of MDA level and SOD, GSH-Px and CAT activities using commercial kits obtained from Nanjing Jiancheng Bioengineering Institute (Jiangsu, China).

### Measurement of lipids parameters in plasma and liver

Plasma TG, TC, LDL-C, HDL-C, and hepatic levels of triglycerides (TG), cholesterol (TC), were measured, respectively, using commercial kits obtained from Nanjing Jiancheng Bioengineering Institute (Jiangsu, China) according to the supplier’s instructions.

### RNA isolation and real-time polymerase chain reaction (RT-PCR)

Total RNA was extracted from liver tissues using a commercial reagent (Invitrogen, CA, USA) according to the manufacture’s protocol. The cDNA synthesis was performed by reverse transcription of 1 µg total RNA using Frist Strand cDNA Synthesis Kit (Thermo, USA). RT-PCR amplification was carried out with an SYBR Green qPCR Master Mix kit (Thermo, USA) according to the manufacturer’s protocol. The qPCR was carried out in duplicate, the condition of RT-PCR amplification reaction as follows: 45 cycles of 95 °C for 10 s, 60 °C for 30 s and 72 °C for 30 s with the primer sequences (Table 1). The expression of target gene transcripts was related to the reference gene (GAPDH). Results were expressed as folds of control.

**Table 1:** Sequences of primers used quantitative real-time PCR

Gene	Forward primer	Reverse primer
SCD-1	5′-TGCTGATCCCCACAATTCCC-3′	5′-CTTTGACGGCTGGGTGTTTG-3′
SREBP-1C	5′-CCCTGCGAAGTGCTCACAA-3′	5′-GCGTTTCTACCACTTCAGGTTTCA-3′
ACC	5′-ACACTGGCTGGCTGGACAG-3′	5′-CACACAACTCCCAACATGGTG-3′
FAS	5′-GGCCACCTCAGTCCTGTTAT-3′	5′-AGGGTCCAGCTGAGGGTACA-3′
GAPDH	5′-GAACGGGAAGCTCACTGGC-3′	5′-GCATGTCAGATCCACAACGG-3’

### The data statistical analysis

All experimental results were reported as the means ± SD. The differences between groups were estimated using one-way ANOVA followed by the Tukey’s multiple comparison test for post-hoc analysis using GraphPad Prism software (GraphPad software, Inc., La Jolla, USA); *P* < 0.05 was usually considered as statistically significant.

## Results

### Component analysis of PUFA in the walnut oil

The PUFA in walnut oil was quantitated by HPLC–UV and qualitative estimation of walnut oil indicated the presence of PUFA about 90.64%. As showed in Table 2, the major fatty acids were linoleic acid (62.47%), followed by oleic acid (15.36%) and linolenic acid (12.81%).

**Table 2:** Composition of PUFA in the walnut oil

Component	Percentage of total fatty acids (%)
Oleic acid (C18:1n9c)	15.36 ± 0.76
Linolenic acid (C18:3n3)	12.81 ± 0.56
Linoleic acid (C18:2n6c)	62.47 ± 1.06

### PUFA improved gestational diabetes mellitus symptoms in pregnant rats

**Fig. 1:**
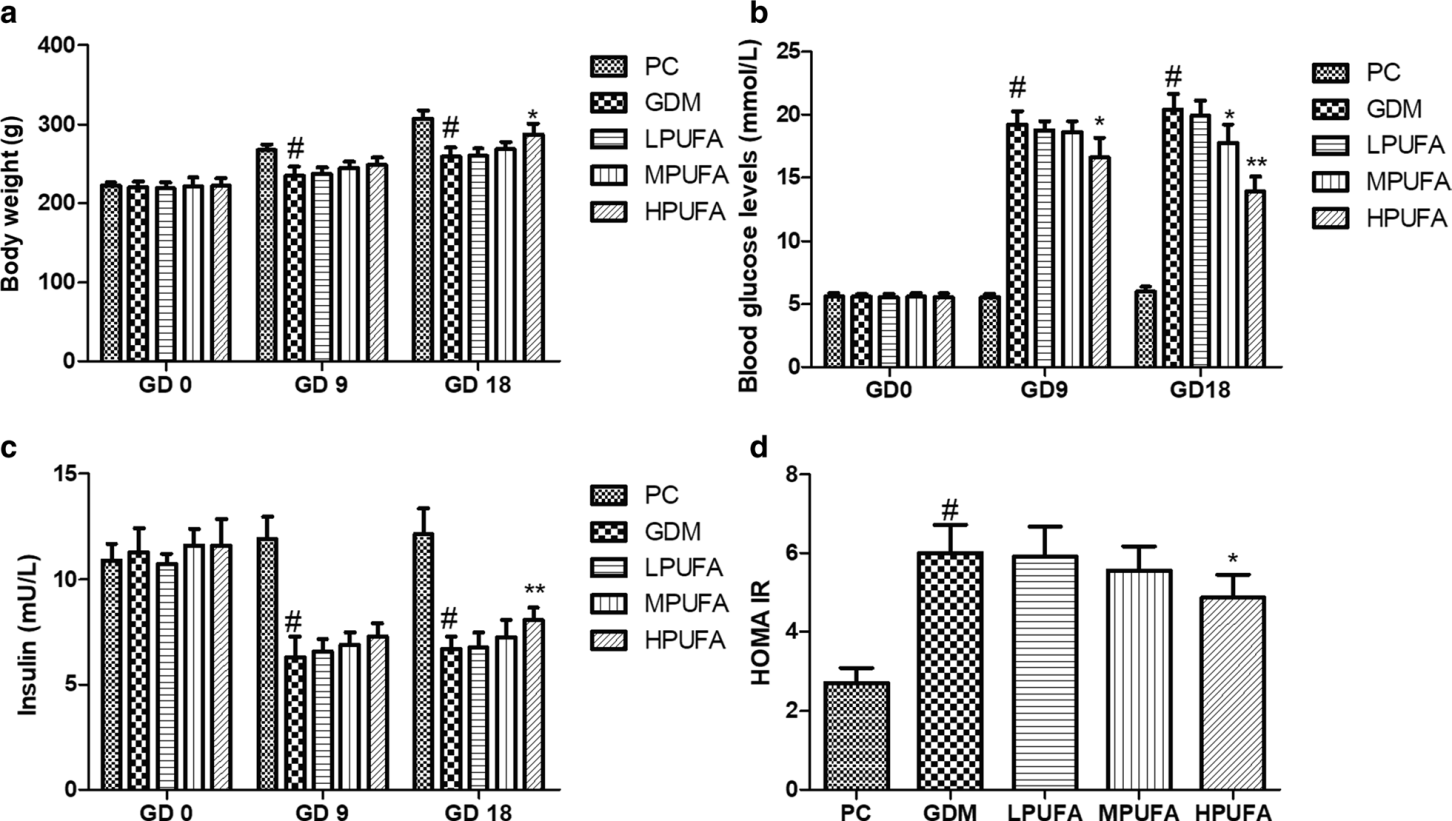
Walnut oil-derived PUFA alleviates gestational diabetes symptoms in GDM rats. Maternal body weight (**a**), blood glucose levels (**b**) and insulin levels (**c**) were measured on gestation day (GD) 0, 9, and 18 in different groups. Homeostatic model assessment of insulin resistant (HOMA-IR) indexes in different groups (D). Data are expressed as the mean ± SD (n = 8/group). ^#^*P* < 0.01 (vs the PC group), ^**^P < 0.01 (vs the GDM group), ^*^P < 0.05 (vs the GDM group)

As shown in Fig. 1a, the maternal body weight in GDM group was decreased compared with that in PC group on GD 9 and GD 18 (*P* < 0.01). The maternal body weight was increased in HPUFA group compared with GMD group (*P* < 0.05). As shown in Fig. 1b, blood glucose level in PC group remained stable on GD 0, GD 9, and GD 18, and the level of blood glucose in GDM group was increased compared with the PC group on GD 9 and GD 18 (*P* < 0.01). However, the blood glucose level was decreased in HPUFA group compared with GMD group (*P* < 0.05 and *P* < 0.01). Similar is shown in Fig. 1c, the level of insulin in GDM group was lower than the PC group on GD 9 and GD 18 (*P* < 0.01), which could be partly imporved by HPUFA treatment (*P* < 0.01). STZ-induced GDM rats exhibited a obvious increase in HOMA-IR when compared to the PC group. However, treatment of HPUFA reduced HOMA-IR index (Fig. 1d).

### PUFA improved glucose and insulin tolerance in pregnant rats with diabetes

**Fig. 2:**
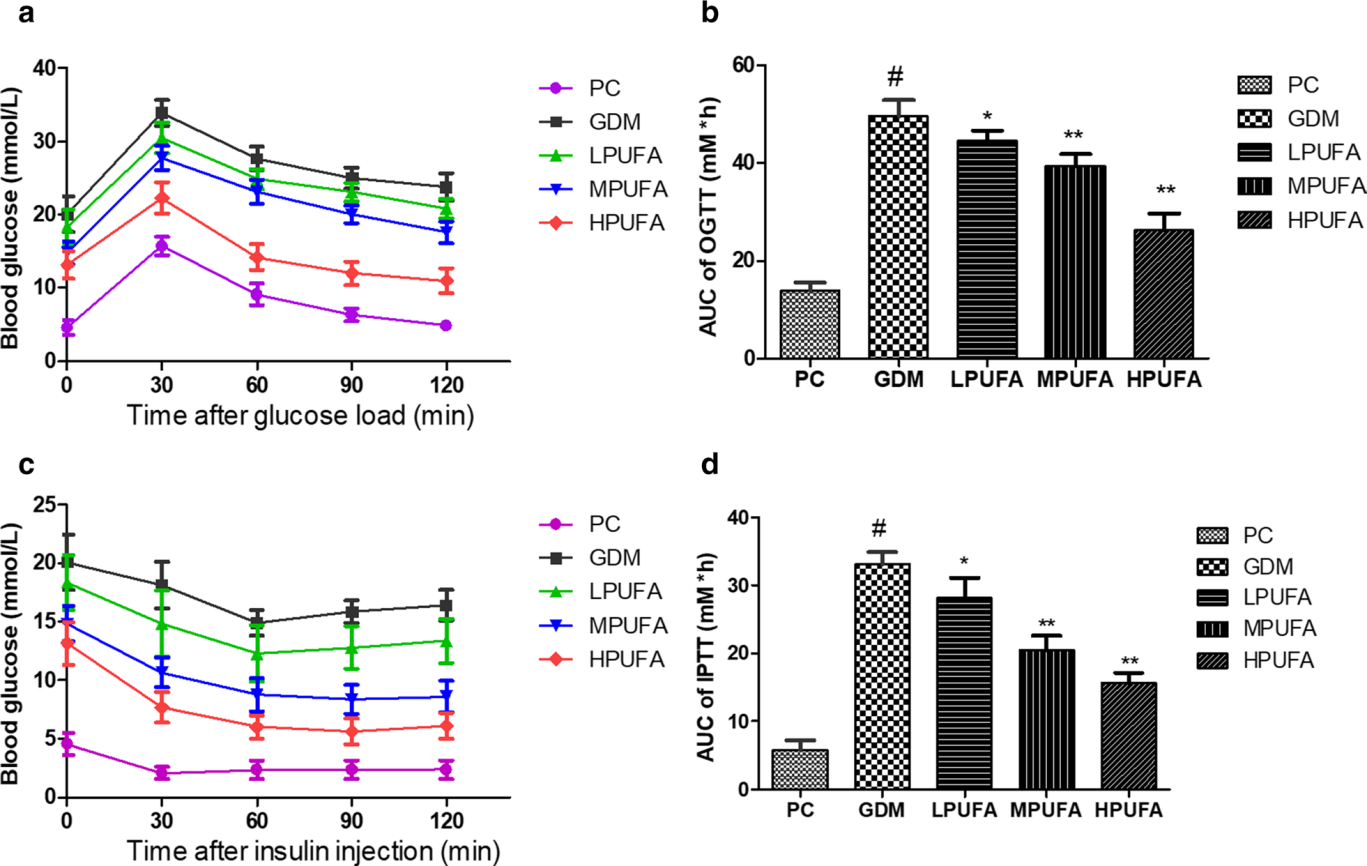
Walnut oil-derived PUFA effectively improves glucose and insulin tolerance in pregnant rats with diabetes. Oral glucose tolerance test (OGTT) and intraperitoneal insulin tolerance test (IPTT) were carried out on gestation day (GD) 17. Effect of PUFA on glucose tolerance (**a**, **b**) and insulin tolerance (**c**, **d**) on GD 17 in pregnant rats with diabetes. Data are expressed as the mean ± SD (n = 8/group). ^#^*P* < 0.01 (vs the PC group), ^**^P < 0.01 (vs the GDM group), ^*^P < 0.05 (vs the GDM group)

The blood glucose levels in all groups were increased after orally administration of glucose. Besides, the levels of blood glucose in the GDM group were always higher than that in the PC group throughout the test (30, 60, 90, and 120 min). However, treatment of HPUFA reduced the glucose level in GDM group (Fig. 2a). Correspondingly, as shown in Fig. 2b, we also observed that PUFA treatment improved glucose tolerance in GDM group measured by glucose area under the curve (AUC) of OGTT (*P* < 0.01). The blood glucose levels in all groups were decreased after injection of insulin. Besides, the levels of blood glucose in the GDM group were always higher than that in the PC group throughout the test (30, 60, 90, and 120 min). However, treatment of HPUFA reduced the glucose level in GDM group (Fig. 2c). Correspondingly, as shown in Fig. 2d, we also observed that PUFA treatment improved insulin tolerance in GDM group measured by AUC of IPTT (*P* < 0.01). Therefore, our findings indicated that treatment of PUFA improved glucose and insulin tolerance in pregnant rats with diabetes.

### Effect of PUFA on the levels of Hb, HbA1c, and liver glycogen in pregnant rats with diabetes

**Fig. 3:**
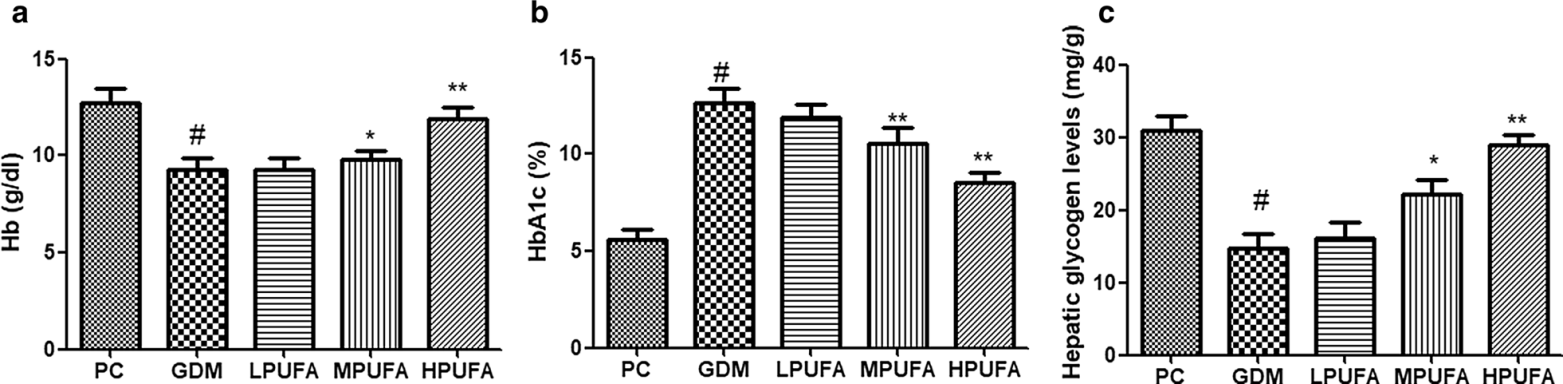
Effect of walnut oil-derived PUFA on the levels of Hb (**a**), HbA1c (**b**), and liver glycogen (**c**) in pregnant rats with diabetes. Data are expressed as the mean ± SD (n = 8/group). ^#^*P* < 0.01 (vs the PC group), ^**^P < 0.01 (vs the GDM group), ^*^P < 0.05 (vs the GDM group)

As shown in Fig. 3, on GD 18, Hb level and hepatic glycogen content of GDM decreased while HbA1c level of GDM group increased in comparison with the PC group (*P* < 0.01). However, the administration of MPUFA and HPUFA to pregnant diabetic rats inhibited the decline of Hb, and hepatic glycogen content and increase of HbA1c level in GDM rats (*P* < 0.05, *P* < 0.01).

### PUFA intervention ameliorates fetal growth restriction and reduces the incidence of embryo lethality under gestational diabetes mellitus condition

**Fig. 4:**
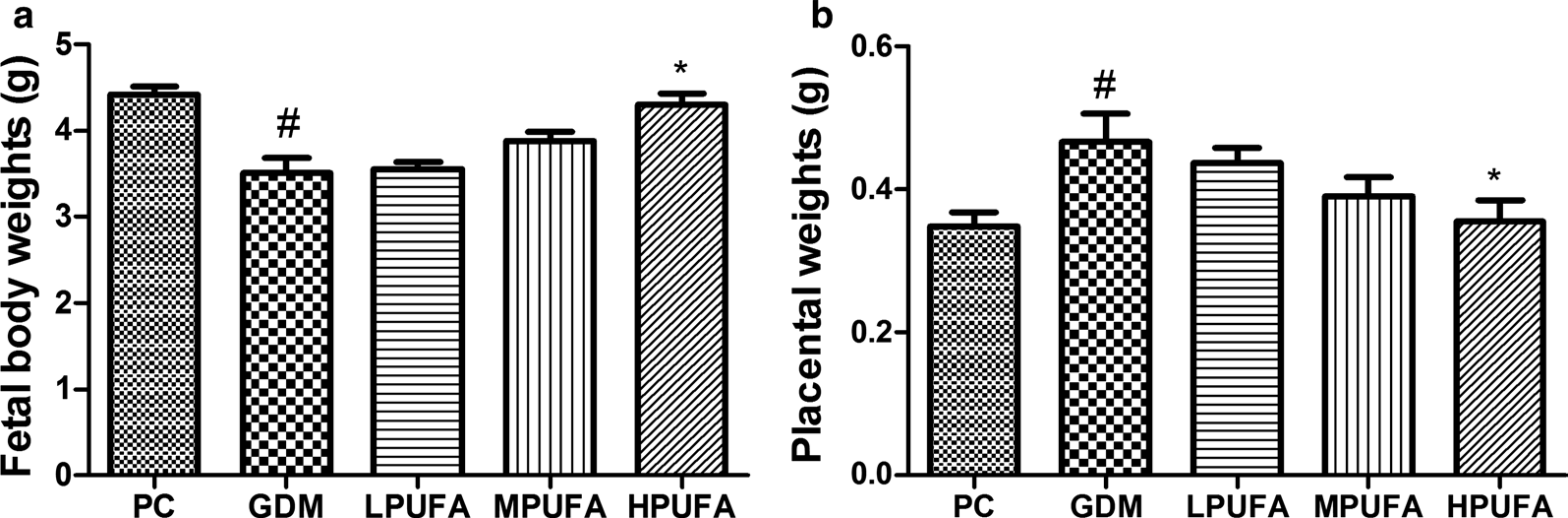
Walnut oil-derived PUFA effectively improves the fetal growth restriction caused by GDM. Fetal body weights (**a**) and placental weights (**b**) were recorded in pregnant rats. Data are expressed as the mean ± SD (n = 8/group). ^#^*P* < 0.01 (vs the PC group), ^*^P < 0.05 (vs the GDM group)

As showed in Fig. 4, compared with the PC group, GDM fetuses suffered an obvious decrease in fetal body weight and increase in the placental weight. However, these characteristics mostly ameliorated by the administration of HPUFA to pregnant diabetic rats (*P* < 0.05). As shown in Table 3, although the incidence of embryo lethality was greater in the GDM group compared with the PC group (*P* < 0.01), PUFA administration of diabetic rats reduced the incidence of dead fetuses (*P* < 0.05, *P* < 0.01).

**Table 3:** Effect of PUFA on the total number of fetuses, the number of live and dead fetuses, and the percentage of dead fetuses in pregnant rats

Group	Total	Live	Dead	% Dead fetuses
PC	104	101	3	2.88
GDM	78	48	30	38.46^#^
LPUFA	82	54	28	34.15
MPUFA	86	62	24	27.91^*^
HPUFA	91	72	19	20.88^**^

The data are expressed as the mean ± SD (n = 8/group)

*PC* pregnant control, *GDM* gestational diabetes model group

^#^*P* < 0.01 (vs. the PC group); ^*^P < 0.05 (vs. the GDM group); ^**^P < 0.01 (vs. the GDM group)

### PUFA intervention ameliorates hepatic oxidative stress under gestational diabetes condition

**Fig. 5:**
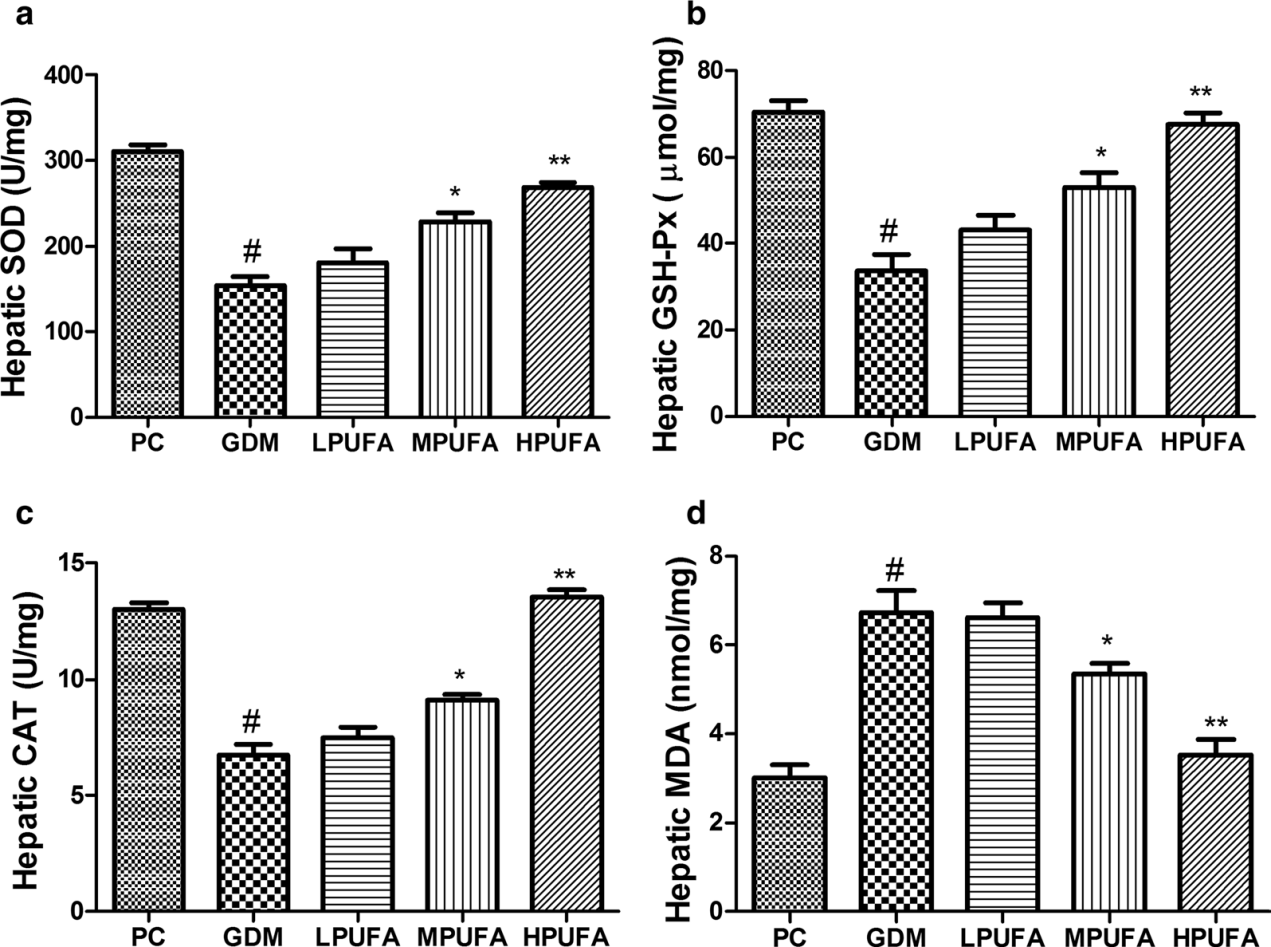
Walnut oil-derived PUFA alleviates oxidative stress in GDM. Hepatic SOD (**a**), hepatic GSH-Px (**b**), hepatic CAT (**c**), hepatic MDA (**d**) were measured in GD 18. Data are expressed as the mean ± SD (n = 8/group). ^#^*P* < 0.01 (vs the PC group), ^**^P < 0.01 (vs the GDM group), ^*^P < 0.05 (vs the GDM group)

As shown in Fig. 5, hepatic SOD, GSH-Px, and CAT activities were significantly decreased while MDA content was increased in the GDM group compared to the PC group (*P* < 0.01). However, oxidative stress ameliorated by the administration of MPUFA or HPUFA to pregnant diabetic rats (*P* < 0.05, *P* < 0.01).

### PUFA intervention ameliorates lipids metabolism disorder under gestational diabetes condition

**Fig. 6:**
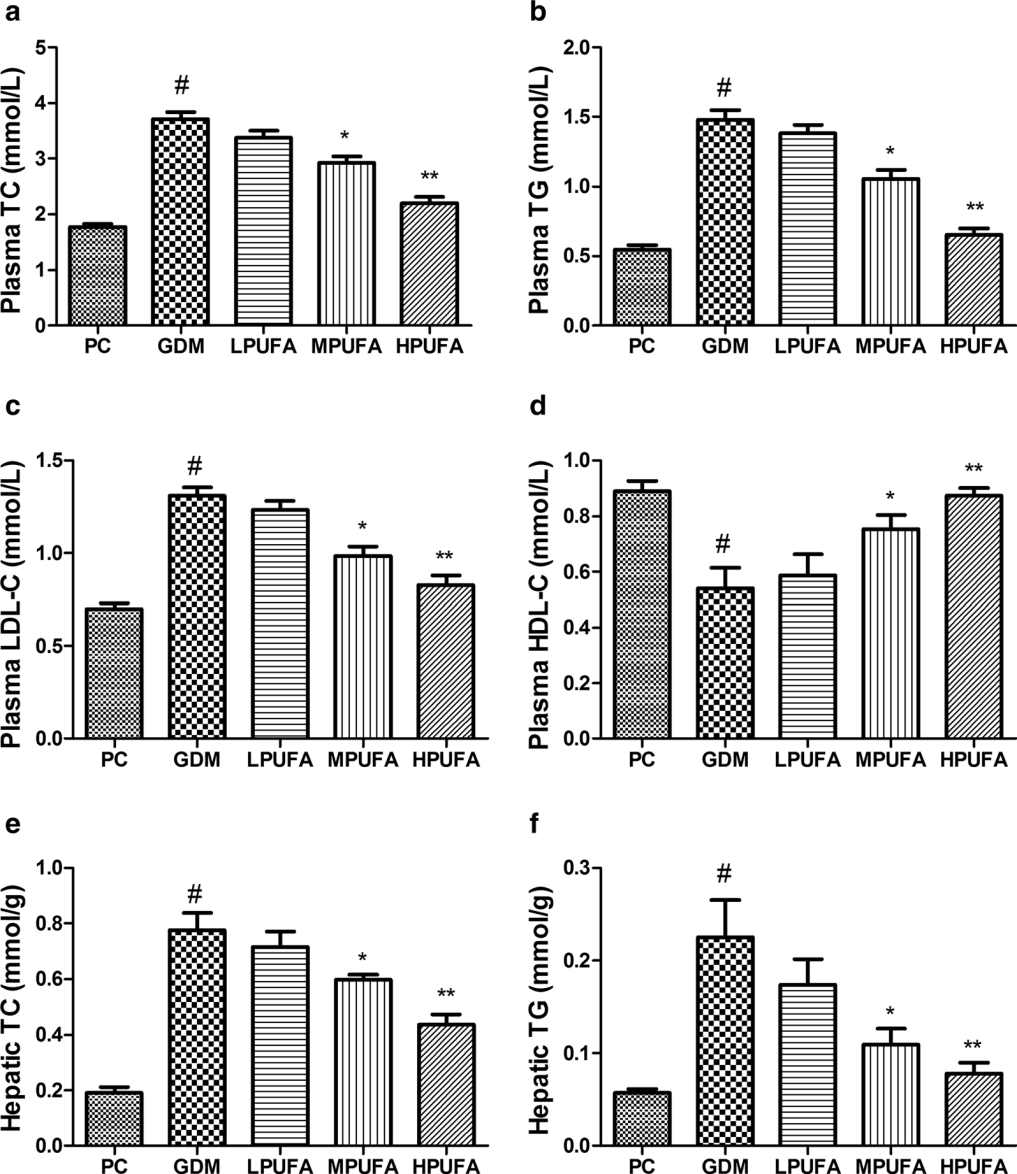
Walnut oil-derived PUFA alleviates lipid metabolism in GDM. Plasma TC levels (**a**), plasma TG levels (**b**), plasma LDL-C levels (**c**), plasma HDL-C levels (**d**), hepatic TC levels (**e**) and hepatic TG levels (**f**) were measured in GD 18. Data are expressed as the mean ± SD (n = 8/group). ^#^*P* < 0.01 (vs the PC group), ^**^P < 0.01 (vs the GDM group), ^*^P < 0.05 (vs the GDM group)

As shown in Fig. 6a–d, plasma levels of TC, TG, and LDL-C were significantly increased while HDL-C was declined in the GDM group compared to the PC group (*P* < 0.01). However, the disorder of lipid metabolism was ameliorated by the administration of MPUFA and HPUFA to pregnant diabetic rats (*P* < 0.05, *P* < 0.01). Besides, the hepatic levels of TC and TG were consistent with the trends in plasma (Fig. 6e,f).

### PUFA intervention suppresses SREBP-1 and its target gene expression under gestational diabetes condition

**Fig. 7:**
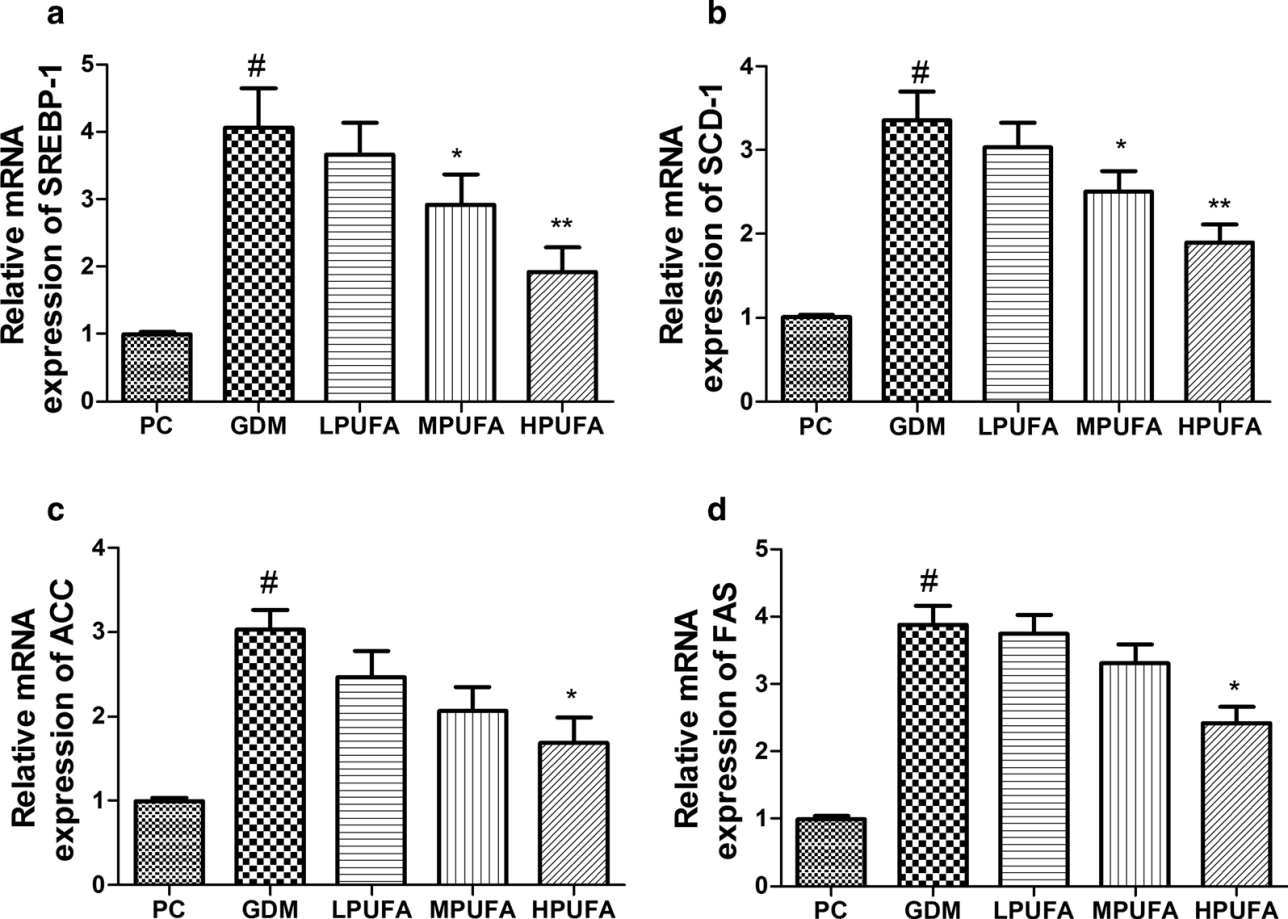
Effect of walnut oil-derived PUFA on the mRNA expression of SREBP-1 and its target genes in pregnant rats. The mRNA expression of SREBP-1 (**a**), SCD-1 (**b**), ACC (**c**) and FAS (**d**) were examined. Data are expressed as the mean ± SD (n = 8/group). ^#^*P* < 0.01 (vs the PC group), ^**^P < 0.01 (vs the GDM group), ^*^P < 0.05 (vs the GDM group)

As shown in Fig. 7, mRNA expression of SREBP-1, SCD-1, ACC and FAS were increased in gestational diabetes rats, compared with those of normal pregnant rats (*P* < 0.01). However, the administration of MPUFA or HPUFA suppressed the increase of SREBP-1, SCD-1, ACC and FAS mRNA expression in gestational diabetes rats of liver tissue (*P* < 0.05, *P* < 0.01).

## Discussion

The use of traditional medicine and food derived from medical plants is increasing in the management of multifarious diabetes-associated complications, largely because of the general notion that traditional medicine and food are less adverse effects compared with synthetic drugs [19]. Recent studies have indicated that a walnut oil-rich diet improved type 2 diabetes [13]. However, its protective effects in STZ-induced diabetes rats during pregnancy have not been investigated so far. In the present study, the typical symptoms of diabetes were induced by the administration of STZ to pregnant rats, such as hyperglycemia and fetal growth restriction, which was consistent with previous studies [17, 20]. What is more, our results indicated that PUFA attenuated gestational diabetes in STZ-induced diabetes rats, as reflected by the decline of fasting blood glucose and the increase of plasma insulin level and hepatic glycogen content. This result was consistent with the previous study of the hypoglycemic effects of flax and sesame seed mixture in diabetic pregnant rats [21].

Previous literature has indicated that experimental induction of gestational diabetes was associated with obvious increases in the incidence of embryo lethality [22, 23]. In the present study, the PUFA administration afforded obvious protection against STZ-induced embryo lethality under gestation diabetic conditions. As far as we know, for the first time, our study investigated the protective effect of PUFA against STZ-induced embryopathy in pregnant rats. Besides, the obvious decline in fetal weight was observed following the STZ-induced GDM. The previous study has reported similar results in STZ-induced GDM, and more importantly, the improvement effects were achieved by the antioxidant *Ipomoea Aquatica* (whole leaf powder) supplementation [17]. This result indicated that the efficacy of antioxidants in ameliorating diabetic embryopathy.

Oxidative stress plays a vital role in the complications of GDM [7]. In the present study, the increased levels of hepatic oxidative stress markers in diabetic rats were in agreement with a previous study [21]. Consistent to previous report [24], our results indicated that PUFA administration counteracted STZ-induced oxidative stress in maternal liver tissue, clearly implying its antioxidative effect in vivo.

It has been reported that abnormal alterations of lipid metabolism were associated with the development of GDM and oxidative stress [25, 26]. And PUFA possessed lipid-lowering activities under different pathological conditions [27, 28]. Consistent with several previous studies [20, 21], our results also showed that GDM induced abnormal changes in lipid metabolism both in plasma and in the liver. These results were connected with an increase in hepatic cholesterol and triglyceride levels, possibly due to increased secretion and synthesis of lipoprotein. In the present study, we observed walnut oil-derived PUFA ameliorated the abnormal changes of lipid metabolism in pregnant rats, as evidenced by the increase of HDL level and the decrease of TG, TC and LDL levels. These findings suggested that PUFA intervention ameliorates lipids metabolism disorder under gestational diabetes condition.

**Fig. 8:**
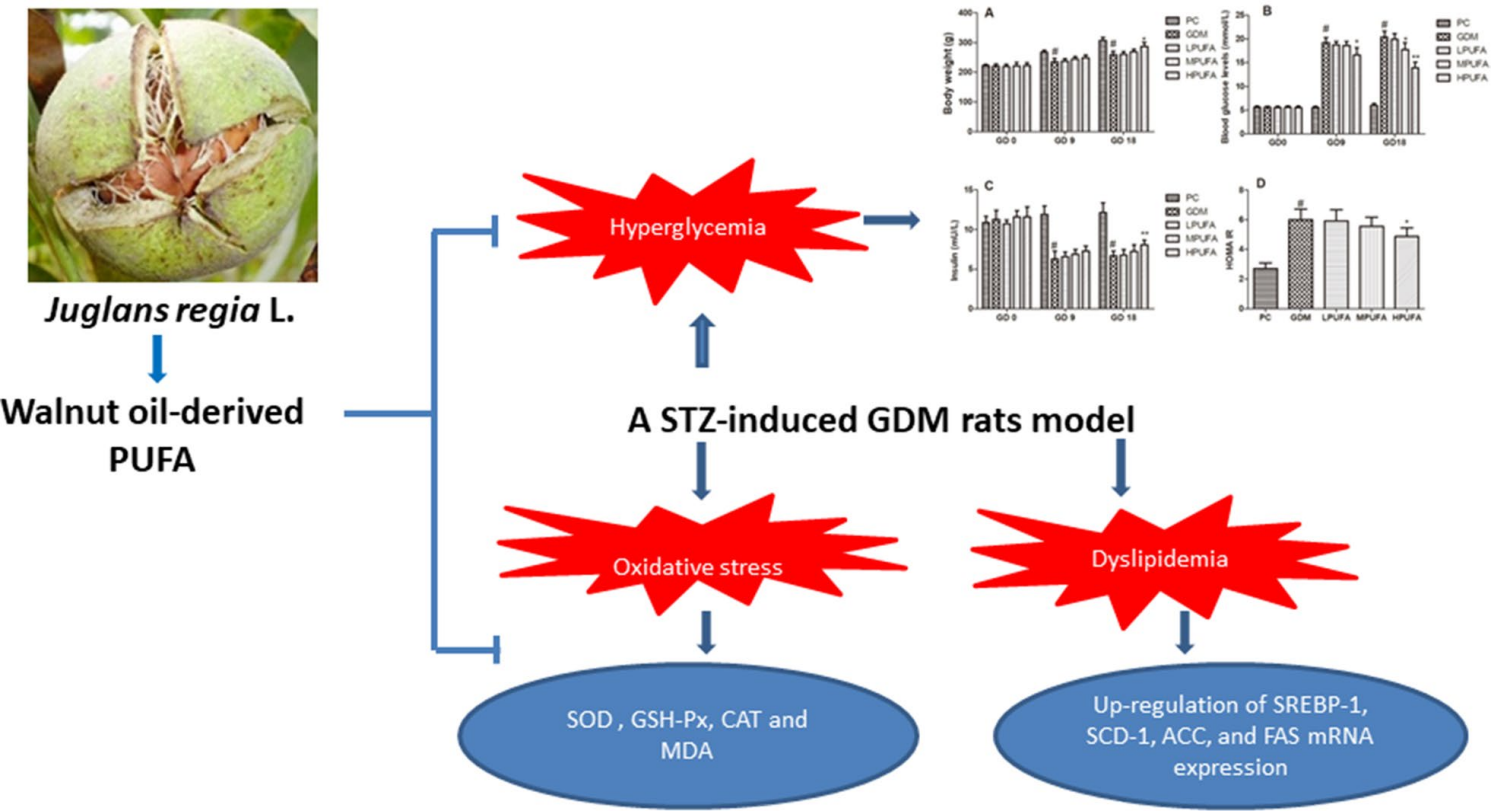
The graphic abstract of the present study. Abbreviations: polyunsaturated fatty acid (PUFA); gestational diabetes mellitus (GDM); gestational day (GD); malondialdehyde (MDA); superoxide dismutase (SOD); catalase (CAT); gutathione peroxidase (GSH-Px); sterol regulatory element-binding transcription factor-1 (SREBP-1); stearoyl-CoA desaturase-1 (SCD-1); streptozotocin (STZ); fatty acid synthase (FAS); and acetyl coenzyme A carboxylase (ACC)

To further investigate the underlying mechanism of PUFA lipid-lowering effect in GDM rats. The gene expressions involved in the fatty acid metabolism in hepatic tissue were examined. SREBP-1 was a nuclear transcription factor played an important role in the regulation of cholesterol, triglyceride, and fatty acids biosynthesis by managing its target genes participated in fatty acid synthesis, including SDC-1, ACC and FAS [29]. Besides, abnormal mRNA expression of SREBP-1 was in connection with the pathogenesis of diabetes [30]. In the present study, we found that PUFA declined SREBP-1, SCD-1, ACC, and FAS mRNA expression in STZ-induced GDM rats. This is attributed to the unsaturated long-chain omega-3 FA and omega-6 could suppress hepatic lipogenesis and promote hepatic fatty acids oxidation, which could improve hepatic insulin sensitivity [31]. Thus, our results showed that PUFA could affect anti-oxidant enzymes activities, and lipid metabolism gene expression that contributes to its beneficial effects on glucose metabolism. The graphic abstract of PUFA prevented hyperlipidemia and oxidant status in pregnant rats with diabetes is showing in Fig. 8.

Although our findings indicated that the beneficial effects of PUFA on GDM, however, there are several limitations that need to be addressed in future investigation. Firstly, the related clinical trial of PUFA safety is limited and although there is no serious side effect of PUFA obserived in pregnant rats model. More safety experiments should be performed in future study. Secondly, only streptozotocin (STZ)-induced GDM rats was used to study GDM. It would be necessary to investigate the beneficial effects of PUFA using other GDM models, such as high-fat diet model and C57BLKsJ^db/+^ mice model. Thirdly, the PUFA is mixture, whether monomers of unsaturated fatty acids have the same hypoglycemic effect remains to be further studied.

## Conclusion

Our results clearly stated that walnut oil-derived PUFA as a therapeutic agent to prevent GDM in pregnant rats. Walnut oil-derived PUFA administration improves the disorders of glucose and lipid metabolism, oxidative stress, and ameliorates insulin resistance in GDM rats, as well as decreased embryo lethality and improved reproductive outcome. Although these findings implied that PUFA has potential become a therapeutic agent for the prevention and treatment of GDM. However, this is a hypothesis-generating study. More animal experiment studies and clearing of the limitations are required before human studies begin.

## Data Availability

The datasets used and/or analyzed during the current study are available from the corresponding author on reasonable request.

## Abbreviations

PUFA: Polyunsaturated fatty acid
GDM: Gestational diabetes mellitus
GD: Gestational day
MDA: Malondialdehyde
SOD: Superoxide dismutase
CAT: Catalase
GSH-Px: Gutathione peroxidase
SREBP-1: Sterol regulatory element-binding transcription factor-1
SCD-1: Stearoyl-CoA desaturase-1
FAS: Fatty acid synthase
ACC: Acetyl coenzyme A carboxylase
TG: Triglycerides
TC: Total cholesterol
LDL-C: Low density lipoprotein cholesterol
HDL-C: High density lipoprotein cholesterol
